# Investigate the Possibility of Using Phosphorescence in Clinical Oncology as an Early Prognostic Test in Detecting Brain Carcinogenesis

**DOI:** 10.1007/s10895-023-03237-9

**Published:** 2023-04-27

**Authors:** Yuriy O. Vinnyk, Igor A. Kryvoruchko, Valeriy V. Boyko, Yulia V. Ivanova, Svetlana Gramatiuk, Karine Sargsyan

**Affiliations:** 1 Department of Oncology, Radiation Therapy, Oncosurgery and Palliative Care, Kharkiv National Medical University, Nauky Avenue, Kharkiv, 61022 Ukraine; 2 Department of Surgery No.2, Kharkiv National Medical University, Nezalezhnosti Avenue, Kharkiv, 61022 Ukraine; 3 Institute General and Emergency Surgery Named After V.T. Zaitcev of the National Academy of Medical Sciences of Ukraine, Balakireva Entry, Kharkiv, 61103 Ukraine; 4 Department of Surgery No.1, Kharkiv National Medical University, Balakireva Entry, Kharkiv, 61103 Ukraine; 5 Institute of Bio-Stem Cell Rehabilitation, Ukraine Association of Biobank, Puskinska Str, Kharkiv, 61022 Ukraine; 6 International Biobanking and Education, Medical University of Graz, Elisabethstraße, 8010 Graz, Austria; 7 Department of Medical Genetics, Yerevan State Medical University, Koryun 30, 0012 Yerevan, Armenia; 8 Cancer Center, Cedars-Sinai Medical Center, Beverly Hills, 90200 USA

**Keywords:** Phosphorescence, Non-invasive glioblastoma testing, L-tryptophan, Cancerous brain tissues

## Abstract

Phosphorescence is considered one of the non-invasive glioblastoma testing methods based on studying molecular energy and the metabolism of L-tryptophan (Trp) through KP, which provides essential information on regulating immunity and neuronal function. This study aimed to conduct a feasibility study using phosphorescence in clinical oncology as an early prognostic test in detecting Glioblastoma. This study was conducted on 1039 patients who were operated on with follow-up between January 1, 2014, and December 1, 2022, and retrospectively evaluated in participating institutions in Ukraine (the Department of Oncology, Radiation Therapy, Oncosurgery, and Palliative Care at the Kharkiv National Medical University). Method of protein phosphorescence detection included two steps. During the first step, of luminol-dependent phosphorescence intensity in serum was carried out after its activation by the light source, according to the spectrofluorimeter method, as follows. At a temperature of 30 °C, serum drops were dried for 20 min to form a solid film. After that, we put the quartz plate with dried serum in a phosphoroscope of luminescent complex and measured the intensity. With the help of Max-Flux Diffraction Optic Parallel Beam Graded Multilayer Monochromator (Rigaku Americas Corporation) following spectral lines as 297, 313, 334, 365, 404, and 434 nm were distinguished and absorbed by serum film in the form of light quantum. The monochromator exit split width was 0.5 mm. Considering the limitations of each of the non-invasive tools currently available, phosphorescence-based diagnostic methods are ideally integrated into the NIGT platform: a non-invasive approach for visualizing a tumor and its main tumor characteristics in the spatial and temporal order. Because trp is present in virtually every cell in the body, these fluorescent and phosphorescent fingerprints can be used to detect cancer in many different organs. Using phosphorescence, it is possible to create predictive models for GBM in both primary and secondary diagnostics. This will assist clinicians in selecting the appropriate treatment option, monitoring treatment, and adapting to the era of patient-centered precision medicine.

## Introduction

Glioblastoma (GBM) is the most aggressive primary brain tumor, with an incidence of 2–3 cases per 100,000 people [1]. The average survival in Ukraine of about 12–14 months is achieved with intensive multimodal treatment, but the number of such patients is minimal. Despite such intensive treatment, there is no cure, and the recurrence of Glioblastoma is inevitable [2].

Approaches to diagnosing GBM are rapidly evolving and are based on the recently revised WHO (2016) criteria for classifying central nervous system tumors [3]. Currently, histopathological examination of a tissue sample for suspected GBM is the gold standard for diagnosis. Now complemented by molecular diagnostics, the identification of O6-methylguanine-DNA methyltransferase (MGMT) methylation, isocitrate dehydrogenase (IDH) mutation, and 1p19q coding is currently the most valuable in daily clinical practice [4].

Several studies have proven that cellular metabolism is the main regulator of tumor behavior [5]. To maintain a high proliferation rate, cancer cells undergo metabolic reprogramming, and tumors use amino acids to meet energy needs and biosynthesis in cells [6]. Tryptophan plays an essential role in malignant conversion and tumor progression. Trp degradation occurs via the kynurenine (Kyn) pathway, followed by the formation of metabolites [7]. Non-invasive glioblastoma testing (NIGT) combines non-invasive (non-surgical) techniques to represent the tumor and provides information about tumor drivers and the microenvironment, all factors that can/should be included in a treatment or diagnostic regimen. Among the large number of NIGTs using spectroscopic methods available for testing cell populations and the functional state of biological fluids, the phosphorescence method occupies a leading position. Important areas of NIGT are radiogenomics, radiomics, and liquid biopsy. The use of serum as a material for the diagnosis and prognosis of glioblastoma in non-invasive methods is, in our opinion, the most relevant, since extracellular vesicles carry molecular components from their parent cells. In the last few years, increasing attention has been paid to liquid biopsy in glioblastoma. Mutation and methylation in serum DNA (cDNA) have been proven to be effective non-invasive tests of glioblastoma for the diagnosis. Circulating tumor cells (CTCs) have also been identified in glioblastoma patients as markers for diagnosis and treatment response.

Phosphorescence spectroscopy of amino acids such as Tryptophan is a fast, easy-to-use, highly sensitive, and highly selective method that allows you to gain in-depth knowledge of the structural, functional, and metabolic changes in cells, organs, and systems that occur at the molecular level [8–10].

Phosphorescence is considered one of the NIGT methods based on studying molecular energy and the metabolism of L-tryptophan (Trp) through the Kynurenine Pathway (KP), which provides essential information on regulating immunity and neuronal function [11–16].

This study aimed to conduct a feasibility study using phosphorescence in clinical oncology as an early prognostic test in detecting Glioblastoma.

## Materials and Methods

### Patient Selection and Data Collection

This study was conducted on 1039 patients who were operated on with follow-up between January 1, 2014, and December 1, 2022, and retrospectively evaluated in participating institutions in Ukraine (the Department of Oncology, Radiation Therapy, Oncosurgery, and Palliative Care at the Kharkiv National Medical University). The respective institutional review boards of each participating institution have approved this study. Demographic and clinical data were collected, including age, gender, concurrent diseases, MRI, white blood cell count, and total neutrophil and lymphocyte count.

This retrospective cohort study was handled in accordance with the Declaration of Helsinki. This manuscript adheres to the applicable STROBE guideline. The use of registered data follows the General Data Protection Regulation of the European Union.

The patient’s written Informed consent was signed for each bio-object from the residual materials. The study and the use of data were consented to by the Ethics Committee of Kharkiv National Medical University, Ukraine (Protocol No. 6, November 11, 2022).

Participants must meet one of the following: diagnosed with a single primary cancer that has not yet been treated. There is no evidence or treatment of any cancer for at least five years before enrollment.

Key Exclusion Criteria: a medical condition which, in the investigator's opinion, should preclude enrollment in the study. Known to be pregnant. Any therapy for cancer, including surgery, chemotherapy, immunotherapy, and/or radiation therapy, in the five years, preceding enrollment. Participated in or is currently participating in a clinical research study in which an experimental medication has been administered in the last 30 days. Participated in or is currently participating in another clinical study.

For the control cohort: Any previous cancer diagnosis in the five years preceding enrollment, recurrence of the same primary cancer within any timeframe, or concurrent diagnosis of multiple primary cancers within any timeframe. The comparative group consisted of 486 conventionally healthy men and women aged from 25 to 65 years.

Serum proteins' phosphorescence state was studied and evaluated in 1039 patients with cancer brains (Glioblastoma), aged from 35 to 68 years. Clinical and histomorphological methods confirmed the diagnosis.

Among 1039 patients were 764 (406 men, 358 women) patients with Glioblastoma (GB) and 275 (150 men and 125 women) with Multiforme Glioblastoma (MGB).

After detecting a brain tumor on CT or MRI, the neurosurgeon obtained tissue from the tumor for biopsy. We used tumor tissue analysis to assign tumor, severity, and histological grade.

The first stage (I) of the disease was diagnosed in 81 patients (these were cases of cystic brain disease), the second (II) in 163, the third (III) in 564, and the fourth stage (IV) was detected in 231 patients.

### Method of protein phosphorescence detection included two steps

During the first step, we obtain a blood sample (5.0 ml) from the ulnar vein and prepare serum by centrifugation using routine methods in medical practice. Centrifugation is used to separate the serum from the blood cells, and then the resulting supernatant is further processed to produce serum aliquots. The centrifugation proceeded in the cooled centrifuge, with a rotation of 1500 × g 10 min.

A study of luminol-dependent phosphorescence intensity in serum was carried out after its activation by the light source, according to the spectrofluorimeter method, as follows: 50 mcl of luminol-containing serum (10 mcl 3% luminol solution) was placed on a 5 × 45 mm quartz plate and placed in a thermostat. At a temperature of 30 °C, serum drops were dried for 20 min to form a solid film. After that, we put the quartz plate with dried serum in a phosphoroscope of luminescent complex and measured the intensity. With the help of Max-Flux Diffraction Optic Parallel Beam Graded Multilayer Monochromator (Rigaku Americas Corporation) following spectral lines as 297, 313, 334, 365, 404, and 434 nm were distinguished and absorbed by serum film in the form of light quantum. The monochromator exit split width was 0.5 mm. The spectral sensitivity of the FEP was located in the ultraviolet and visible ranges of daylight. Coating technology provides results in less than 0.5% d-spacing variation.

In the second stage, we examined the tumor tissue after surgery. Fresh surgical specimens of cancerous brain tissues were acquired under Ukraine Association of Biobank approval from a tissue bank within three h of surgery. The standard tissue specimens were from the tumor's margins and were a mix of malignant and normal tissues. Samples were stored at 4 °C and not subjected to additional processing before measuring the spectra. Specimens were cut into pieces ranging from ∼0.5 to ∼1 cm^2^ and placed in a 1- × 1-cm quartz cuvette before mounting on the translation stage. Using the spectrograph setup, the spectra were acquired in a grid pattern with a 0.5—to 1-mm spacing to cover most of the specimen. Each spectrum was integrated for 2 s. Intensity ratio maps were generated from the individual spectra.

### Statistical Analysis

The research materials were subjected to statistical processing using the methods of parametric analysis. Accumulation, correction, systematization of initial information and visualization of the obtained results were carried out in Microsoft Office Excel 2016 spreadsheets. Statistical analysis was carried out using the software package STATISTICA 13.3 (StatSoft.Inc).

In the case of describing quantitative indicators with a normal distribution, the obtained data were combined into variational series, in which the arithmetic means (M) and standard deviations (SD) were calculated, the boundaries of the 95% confidence interval (95% CI) for the difference were determined. Sets of quantitative indicators, the distribution of which differed from normal, were described using the values of the median (Me) and the lower and upper quartiles (Q1-Q3). When comparing mean values in normally distributed sets of quantitative data, Student's t-test was calculated. The obtained values of the Student's t-test were evaluated by comparison with critical values. Differences in indicators were considered statistically significant at a significance level of p < 0.05 [17].

## Results

The highest level of phosphorescence was observed at activation with a 297 nm spectral wave. In this case, the levels of phosphorescence intensity of blood serum in patients with MGB and GB upon activation at 297 nm increased on average by 86.2% and 109.7%, respectively, concerning the indicators of conditionally healthy people. The intensity of serum phosphorescence at activation with the spectral line of 404 nm was increased by 3.7 and 3.6 times, or by 271.4% and 258.6%, respectively, in patients with GB and MGB compared to a group of conditionally healthy people. The levels of phosphorescence intensity of blood serum in patients with MGB and GB upon activation at 404 nm by 274.9% and 259.7%, and at 434 nm, 207.7% and 226.6%, respectively. Wherein, the more negligible difference in phosphorescence was observed at activation with spectra of 313 nm, 334 nm, and 365 nm: at 313 nm, phosphorescence intensity was raised by 29.2% and 52.1%; at 334 nm, by 26.7% and 21.7%; and at 365 nm, by 67.2% and 62.9% in patients with GB and MGB, respectively (Table 1).

**Table 1:** The intensity of luminol-dependent serum phosphorescence in patients with brain cancer and conditionally healthy people depends on tumour type

The range of activation (nm)	Phosphorescence intensity ( I^0^c), M ± SD; Me, IQR			**P-value**
	Patients with Glioblastoma (n = 1039)		Conditionally healthy people (n = 486)	
	MGB (n = 275)	GB (n = 764)		
297	5979,3 ± 188,7*	6731,4 ± 144,2*	3210,7 ± 137,5	0.000
	6011,2 (5789,1–6021,4)	6738,7 (6632,5–6892,8)		
	**χ2/P-value**: 164.815∕0.000			
313	437,6 ± 5,163*	483,5 ± 8,96*	317,8 ± 19,6	0.000
	437,6 (432,4–443,4)	482,2 (481,9–485,1)		
	**χ2/P-value:** 92.11∕0.000			
334	807,4 ± 5,989*	796,3 ± 13,94*	653,9 ± 39,6	0.000
	809,4 (792,3–812,8)	797,7 (781,8–811,2)		
	**χ2/P-value:** 147.738∕0.000			
365	3014,5 ± 39,08*	2868,7 ± 54,3*	1760,5 ± 59,2	0.000
	3012,4 (3008,5–3022,7)	2869,3 (2843,7–2883,2)		
	**χ2/P-value:** 92.529∕0.000			
404	1894,3 ± 24,39*	1812,4 ± 39,6*	505,3 ± 39,4	0.000
	1896,6 (1874,3–1995,7)	1812,9 (1802,6–1819,4)		
	**χ2/P-value:** 197.531∕0.000			
434	1826,4 ± 34,23*	1938,6 ± 25,2*	593,6 ± 30,9	0.000
	1829,3 (1819,2–1833,4)	1940,7 (1931,3–1944,2)		
	**χ2/P-value:** 159.727∕0.000			

^*^ − statistically significant indicators at a significance level of p < 0.05 compared to conditionally healthy people

The study of serum phosphorescence in GB patients revealed a high informativeness of indexes at activation with a spectral range of 297 nm, 404 nm, and 434 nm. It revealed a direct correlation between the degree of disease severity and the intensity of phosphorescence in GB patients' serum (Table 2).

**Table 2:** The intensity of luminol-dependent serum phosphorescence in patients depending on the stage of GB and MGB

The range of activation (nm)	Phosphorescence intensity ( I^0^c), M ± SD					
	Stage of GB and MGB, n				Conditionally healthy people (n = 486)	95% CI ∕ P -value
	Stage—I n = 81	Stage – II n = 163	Stage – III n = 564	Stage – IV n = 231		
297	5706,3 ± 127,5*	6157,8 ± 104,5*	6508,3 ± 118,6*	6837,4 ± 162,7*	3210,7 ± 137,5	3211 to 3237∕ 0.000
313	394,8 ± 3,62*	415,6 ± 3,72*	488,9 ± 4,76*	520,6 ± 4,86*	317,8 ± 19,6	133.9 to 140.1∕ 0.000
334	762,5 ± 13,8*	794,3 ± 16,5*	825,6 ± 5,2*	863,7 ± 19,4*	653,9 ± 39,6	154.1 to 161.9∕ 0.000
365	2796,8 ± 35,3*	2837,6 ± 33,4*	2925,8 ± 47,4*	3097,6 ± 55,8*	1760,5 ± 59,2	11.46 to 11.62∕ 0.000
404	1754,6 ± 26,3*	1814,3 ± 28,8*	1897,2 ± 33,6*	1972,4 ± 43,5	505,3 ± 39,4	1348 to 1360∕ 0.000
434	1710,3 ± 34,5*	1794,8 ± 23,6*	1923,8 ± 25,4*	1986,7 ± 28,3*	593,6 ± 30,9	1254 to 1266∕ 0.000

^*^ − statistically significant indicators at a significance level of p < 0.05 compared to conditionally healthy people

**Fig. 1:**
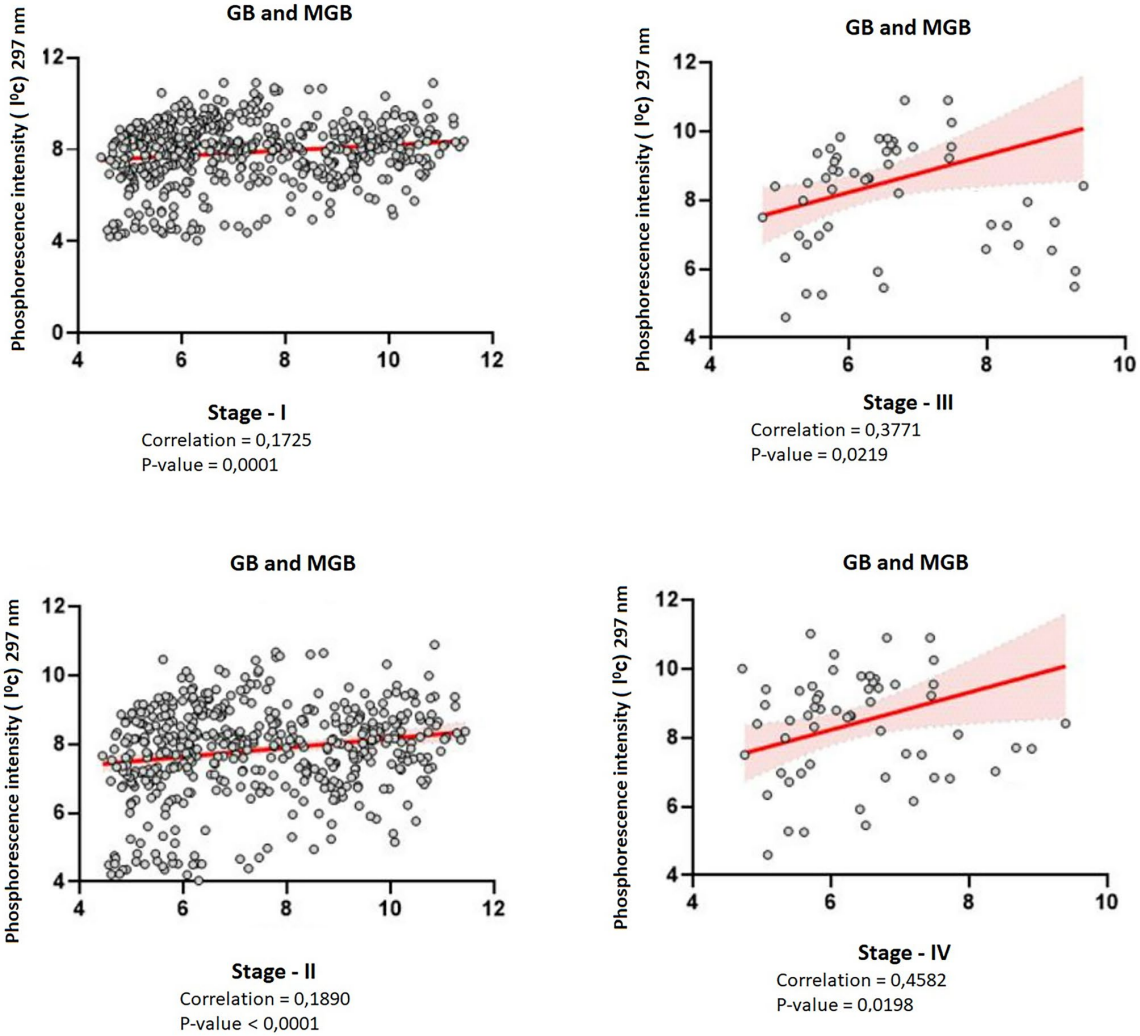
Correlation of phosphorescence and stage of glioblastoma upon activation by a spectral wave of 297 nm

Correlations between diagnostic tools include magnetic resonance imaging and serum phosphorescense Expression in GBM. To analyze whether the serum phosphorescense expression correlates with Glioblastoma stages, we performed correlation analysis across samples registered with Ukraine Association of Biobank dataset. We detected a weak correlation between of phosphorescence was observed at activation with a 297 nm spectral wave and stage I when considering GBM samples together (Fig. 1A, correlation = 0.1725, p = 0.0001) and stage II GBM samples (Fig. 1B, correlation = 0.1890, p ≤ 0.0001). A moderate correlation between of phosphorescence was observed at activation with a 297 nm spectral wave and Stage III glioblastoma (Fig. 1C, correlation = 0.3771, p = 0.0219), and Stage IV (Fig. 1D, correlation = 0.4582, p = 0.0198) higt correlation between of phosphorescence and wiht stage IV glioblastoma.

Inactivation of serum with the lower ultraviolet (365 nm) phosphorescence increased by more than 60% compared to a group of conditionally healthy people. In all groups and at different spectral lines, a correlation between phosphorescence intensity and the stage of the tumor process took place.

**Fig. 2:**
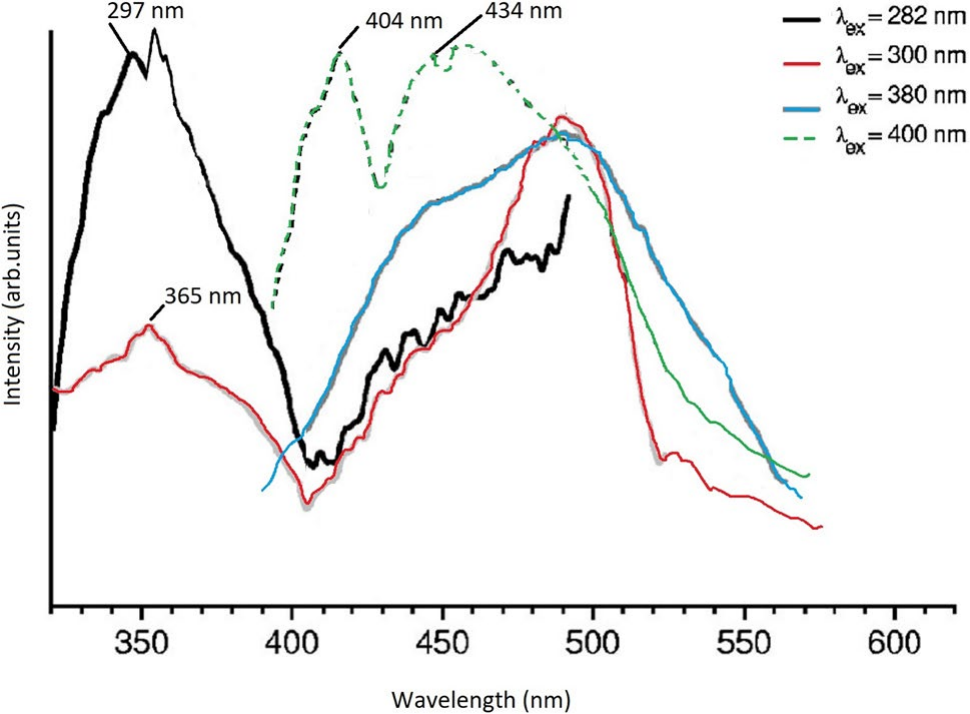
Phosphorescence spectra of D-L tryptophan powder for different excitation wavelengths cancerous brain tissues. Spectra are integrated from 0.5- to 7.5-ms delay concerning the excitation lamp pulse

The phosphorescence spectra of D-L-trp powder excited at 282, 300, 380, and 400 nm and acquired with the CD-Scan cancerous brain tissues are shown in Fig. 2. Each spectrum shown in the figure is the sum of seven ranges, obtained with delays of 1 to 7 ms in 1-ms steps.

The gate width was 1 ms. Thus, each range shown is the integrated intensity from t = 0.5 to 7.5 ms. Although it is well known that the trp excitation maximum is at 282 nm and that trp does not have significant absorption at wavelengths longer than 300 nm, the phosphorescence intensity with 282 nm excitation was weaker than with 400 nm excitation. The phosphorescence was blue-shifted for the longer wavelength excitations (380 and 400 nm) compared to the shorter wavelength excitations (282 and 300 nm). For 380 nm and 400 nm excitation, trp exhibits two phosphorescence peaks at 480 nm and 525 nm. For excitation at 282 nm and 300 nm, only the phosphorescence peak at 525 nm was observed. The mechanism responsible for the shift in phosphorescence and the reason for the more intense at 400 nm excitation is unknown. It may be due to dimers or trimers present in the trp powder.

**Fig. 3:**
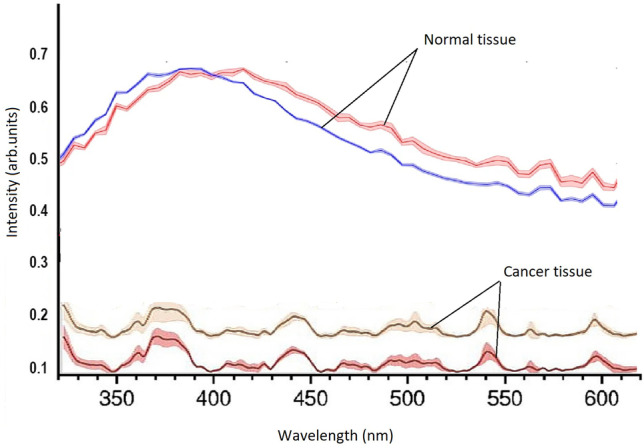
Phosphorescence from normal and malignant brain tissues. Signals were integrated for detector gate delays of 1 to 7 ms

*Phosphorescence from *ex vivo* human brain tissues*. Although the phosphorescence intensity in trp powder was greater for excitation at 400 nm than for excitation at 300 nm, tissues contain several fluorophores (NADH and flavins) whose emission wavelengths can overlap with the trp. Therefore, the brain tissue specimens were excited at 300 nm to reduce the contribution from these fluorophores. Figure 3 shows the phosphorescence from ex vivo human normal and malignant brain tissues acquired with the CD-Scan. The plots shown in the figure are each the integration of seven spectra obtained with gate delays of 1 to 7 ms with 1-ms intervals. The normal tissues exhibited phosphorescence emission from 440 to 500 nm. Fluorescence can also be observed at 350 nm — most likely due to a long “tail” on the lamp emission. The normal tissues showed more excellent fluorescence and phosphorescence than the malignant tissues, which had almost no detectable phosphorescence.

## Discussion

Optical biopsy—using native tissue fluorescence—for detecting cancer has been an area of investigation for over two decades. The bases behind the operation of optical biopsy are that the onset of carcinogenesis results in structural changes (thickening of the mucosa layer, increased vascularity) and molecular changes (increased nucleic acids, alterations in protein structure, increased cell metabolism) that modify the spectroscopic properties of tissue and, thereby, create unique optical signatures that can be used to detect malignant and premalignant tissues. In the UV and blue spectral regions, the primary native tissue fluorophores are Tryptophan (trp), collagen, elastin, reduced nicotinamide adenine dinucleotide (NADH), and flavins [18, 19]. Optical biopsy has been demonstrated to be an accurate, real-time tool for distinguishing normal tissues from malignant and premalignant tissues. Prior investigations have demonstrated that several ratio-based algorithms can distinguish malignant tissue from normal tissue with high sensitivity and specificity. The wavelength combinations used in those ratios isolated the contributions from different pairs of tissue fluorophores, one of which was trp. The ratio of 340 nm to 440 nm emission (with 300 nm excitation) is useful for identifying malignant tissues from many different organ sites [20].

According to the findings, the appearance of many molecules in the triplet state in the long-wavelength spectral region of activation may indicate a dissociation of oxidative phosphorylation. The tissue respiration, inhibition of bioenergy processes, which are accompanied by ineffective use of energy and scattering it in the form of heat, and reduced ATP production are symptoms of mitochondrial pathology [21–24]. The presence of high energy levels in activated electronic states due to the appearance of unpaired electrons in the active molecule indicates a change in the reaction ability and conformational properties of proteins, nucleic acids, and other biologically active molecules (enzymes, hormones). It is known that ultraviolet spectrum photons are absorbed mainly by aromatic amino acids (tyrosine—at 280 nm, Tryptophan—at 220 nm), proteins (at 280–300 nm), nucleic acids, and nucleotides (at 260 nm) which may be present in the serum. An analysis of the results reveals that profound metabolic, structural, and conformational changes of large polymer molecules and their monomeric components occurred in patients with brain cancer. Increased serum phosphorescence intensity in the long-wave region (404–434 nm) may indicate an increase in non-erythrocytic hemoglobin levels and violations in its compact structure, conformational properties, and hemin content (404 nm) as a result. The development of free radical-membrane pathology in patients with brain cancer underpins the formation of hypochromic anemia in carcinogenesis.

The study of serum phosphorescence intensity in patients with MGB and MG detected violations of the structural and conformational properties of biologically important macromolecules (proteins, nucleic acids, hemoglobin, and other glycoproteins). The presence of many electron-activated molecules, which can stimulate free radical processes and disconnect oxidative phosphorylation, causes energy deficiency, tissue hypoxia, and membrane molecular pathology, all of which are pathogenic factors in brain carcinogenesis [25, 26].

Levels of luminol-dependent serum phosphorescence intensity at activation with 297, 404, and 434 nm can be used to diagnose disease severity and the state of bioenergy processes and plan surgery and pathogenetic therapy. Such spectral monochromatic activation waves as 297, 404, and 434 nm are the most informative. They indicate a disturbance in serum proteins' compact structure and conformational properties and decreased biological activity. High levels of phosphorescence intensity in the first stage of carcinogenesis show changes in the compact design and biological activity of proteins for quite a long period, which precedes the development of the tumor process and is a prognostic factor in the development of oncopathology and its early diagnostic criteria [12–15].

Prolonged activation of oxidative processes leads to changes in protein conformation and biological activity observed in patients with brain cancer. The appearance in the long-wavelength spectral region of activation (404 and 434 nm) and the increased number of molecules in the triplet state may also indicate disconnection of oxidative phosphorylation and tissue respiration, which is always accompanied by heat energy scattering and mitochondrial pathology development, which are pathogenetic factors in carcinogenesis. Increased serum phosphorescence intensity in the ultraviolet region of 297 nm indicates the presence of high levels of triplet-activated states. It shows the change in proteins and nucleic acids' compact structures. The increase in serum phosphorescence in brain cancer patients in the long-wave spectral region of activation (404 nm) reflects an increase in free hemins (a nonprotein component of hemoglobin), confirming the protein's loss of compact structure and biological activity.

Informative monochromatic spectral waves of activation in evaluating serum compact structure and biological activity were 297, 404, and 434 nm. Physiological indexes of luminol-dependent serum phosphorescence intensity at activation with 297 nm range from 3000 to 3500 imp/sec. Increasing luminol-dependent serum phosphorescence intensity at activation with 404 nm from 3500 to 5000 and with 434 nm from 400 to 1700 imp/s indicates a conformational change in the structure and biological activity of serum proteins; this can be a significant prognostic indicator in the early diagnosis of the precancerous metabolic state that leads to carcinogenesis and stage-dependent pathological processes. A further rise in luminol-dependent serum phosphorescence intensity from 5000 to 7000 imp/s at 297 nm and from 1700 to 2000 imp/s at 404 and 434 nm indicates an increase in the severity of the disease.

This newly developed optical technique for cancer detection, based on phosphorescence and fluorescence spectroscopy, is fast, minimally invasive, and non-destructive. This technique may apply to in vivo or ex vivo tissue analysis. The 345 ∕ 500 ratio, with excitation at 300 nm, provides an excellent fingerprint for cancer detection in ex vivo tissues. A 345 ∕ 500 ratio of 0.5 to 12 corresponds to normal brain tissue, and a ratio higher than 12 indicates cancerous brain tissue. Using ratios as markers for malignancy enables the comparison of data under different illumination conditions and with different surface structures.

Detection of protein aggregation, loss of its compact structure, increasing macromolecule rigidity, and loss of serum biological activity, in combination with the activation of serum phosphorescence may play a decisive role in non-invasive glioblastoma testing.

## Conclusions

Considering the limitations of each of the non-invasive tools currently available, phosphorescence-based diagnostic methods are ideally integrated into the NIGT platform: a non-invasive approach for visualizing a tumor and its main tumor characteristics in the spatial and temporal order. Because trp is present in virtually every cell in the body, these fluorescent and phosphorescent fingerprints can be used to detect cancer in many different organs. Using phosphorescence, it is possible to create predictive models for GBM in both primary and secondary diagnostics. This will assist clinicians in selecting the appropriate treatment option, monitoring treatment, and adapting to the era of patient-centered precision medicine.

Further studies will be required to confirm that the methods used here will provide accurate tissue diagnostic information in vivo.

## Data Availability

The datasets generated and/or analyzed during the current study are available from the corresponding author on reasonable request.

## Abbreviations

CHP: Conditionally healthy people
CT: Computed tomography
GB: Glioblastoma
IDH: Isocitrate dehydrogenase
Kyn: Kynurenine
KP: Kynurenine Pathway
MGB: Multiforme Glioblastome
MGMT: O6-methylguanine-DNA methyltransferase
MRI: Magnetic resonance imaging
NADH: Nicotinamide adenine dinucleotide
NIGT: Non-invasive glioblastoma testing
Trp: L-tryptophan
